# Validating the crèche educator emotional style questionnaire among Chinese kindergarten teachers

**DOI:** 10.3389/fpsyg.2025.1552871

**Published:** 2025-04-08

**Authors:** Biru Chang, Qiuxia Guo, Xingxing Wu

**Affiliations:** ^1^School of Education Science, Nanjing Normal University, Nanjing, Jiangsu, China; ^2^School of Pre-school Education, Xi’an University, Xi’an, Shaanxi, China; ^3^School of Teacher Education, Shanwei Institute of Technology, Shanwei, Guangdong, China; ^4^Nanjing Yixian Experimental Kindergarten, Nanjing, Jiangsu, China

**Keywords:** meta-emotion philosophy, CEESQ, kindergarten teachers, validation study, China

## Abstract

**Introduction:**

Kindergarten teachers are important emotional socialization agents for preschoolers’ social–emotional competence, and their meta-emotion philosophy might either enhance or inhibit preschoolers’ emotional socialization. Due to the lack of relative measurements, no studies have paid much attention to Chinese kindergarten teachers’ meta-emotion philosophy.

**Methods:**

The present study included multistage research to evaluate the psychometric properties of the Crèche Educator Emotional Style Questionnaire (CEESQ) among Chinese kindergarten teachers. In Study One, exploratory factor analyses (EFAs) were conducted to assess the factor structure among 535 Chinese preschool teachers. In Study Two, confirmatory factor analyses (CFAs) were performed to confirm the factor structures among 538 other Chinese preschool teachers. The measurement invariance and convergent validity of the Chinese versions of CEESQ were also assessed. The results indicated that the five-factor model was a feasible representation of the Chinese version of CEESQ factor structure (*χ^2^/df* = 3.70, CFI = 0.93, TLI = 0.92, RMSEA [90% CI] = 0.061 [0.057, 0.065], RMSEA = 0.05). A multigroup CFA indicated the measurement invariance considering teachers’ parenting experiences, amount of teaching experiences, and academic education level.

**Results:**

The Chinese version of CEESQ had robust internal consistency reliability and concurrent validity with emotional intelligence. The Chinese version of CEESQ had sound psychometric characteristics to evaluate Chinese kindergarten teachers’ meta-emotion philosophy.

**Conclusion:**

This study added to the existing research literature by supporting the applicability of CEESQ among Chinese kindergarten teachers.

## Introduction

Increasing research has demonstrated that preschoolers’ social–emotional competence is essential for facilitating their social skills, school success, and mental health, with long-lasting effects on adulthood (Denham, 2023; Denham and Brown, 2010; Jones et al., 2015; Zhu et al., 2024). Therefore, it is essential to explore the factors that may facilitate the development of preschoolers’ social–emotional competence. Both parents and teachers are regarded as significant socializers of emotion, and their meta-emotion philosophy might either enhance or inhibit preschoolers’ social–emotional competence. The meta-emotion philosophy of kindergarten teachers in fostering young children’s emotional competence has consistently been overlooked in comparison to that of parents. The primary explanation may be because the concept of meta-emotion philosophy, initially presented by Gottman et al. (1996), had only been referred to parents and not to other meaningful socializers, such as teachers. Given that teachers perform many emotion-laden care-giving tasks and work as socializers of children’s social–emotional competence by modeling, scaffolding, or reacting to their emotions as much as parents, Ciucci et al. (2015) and Ciucci and Baroncelli (2024) first extended the construct of parental meta-emotion philosophy, respectively, to crèche educators and to teachers samples. They subsequently developed the Crèche Educator Emotional Style Questionnaire (CEESQ).

Research on the CEESQ has demonstrated its sound psychometric properties, distinct individual characteristic variations, significant inter-relationships, and substantial connections to teachers’ emotion beliefs, mind-mindedness, and emotion socialization practices (Ciucci and Baroncelli, 2024; Ciucci et al., 2015; Ornaghi et al., 2019; Ornaghi et al., 2021). Nonetheless, the majority of these data originated from Italian teachers, thus constraining the applicability to diverse cultural contexts. Instead of openly expressing emotion, individuals from the collectivist culture may prefer to express and regulate their emotions through spiritual practices, allowing them to process difficult feelings and seek solace without disturbing their loved ones (Daga et al., 2015; Merchant, 2024). These emotional behaviors may reflect differences in emotional beliefs (e.g., meta-emotion philosophy) between individuals in various cultural backgrounds. In Chinese societies, a typical representative of collectivist culture, individuals frequently prioritize emotional tolerance and regulation. When they educate their children, they always tend to socialize their children’s emotions by promoting children’s emotional self-regulation, limiting children’s expression of negative emotions, and developing children’s high levels of self-control (Mesquita and Frijda, 1992). Therefore, new insights might be found regarding teachers’ meta-emotion philosophy by examining the reliability and validity of the CEESQ within a Chinese teacher sample.

### Parental meta-emotion philosophy and its effects on children’s social–emotional development

The concept of parental meta-emotion philosophy was first introduced by Gottman et al. (1996) as parents’ thoughts and feelings about their own emotions and the emotions of their children. Meta-emotion philosophy involves an organized set of feelings and thoughts (i.e., A philosophy of emotional expression and regulation) about their own and their children’s emotions, as well as their approach to managing these emotions (Gottman et al., 1996). Specifically, there are two main styles of meta-emotion philosophy: coaching style and dismissing style. Coaching refers to a style in which caregivers are often aware of both their own and their children’s feelings, empathetically accept these emotions, and adeptly assist children in understanding and managing emotions. In contrast, dismissing refers to a style in which caregivers often lack awareness of both their own and their children’s emotions and fail to accept and support children’s negative emotions effectively. Coaching style was positively correlated to children’s social–emotional competence (Liang et al., 2011). Recently, two review studies comprehensively demonstrated the correlation between parental meta-emotion philosophy and children’s social–emotional competence (Katz et al., 2012; Merchant, 2024). Both asserted that coaching styles have been linked to greater child awareness, acceptance, understanding, and regulation of emotion, all of these being key component of children’s social–emotional competence.

Given that the family is essential for children’s emotional socialization processes, above all in childhood (Eisenberg et al., 1998), parental meta-emotion philosophy has attracted much attention, emphasizing that parental beliefs, thoughts, and attitudes about emotions constitute an underlying basis for children’s social–emotional competence. Parents, with coaching style, would give children more accepting and warm emotional responses to their negative emotional reactions, calmly discuss the causes of negative emotions with them, and teach them to cope with negative emotions in a more positive way in daily life, thereby facilitating their emotional competence. Among 334 parents with a child aged five to seven, Qu et al. (2011) found parental coaching style could significantly predict children’s emotion regulation. With a longitudinal approach, Frogley et al. (2023) discovered that the dismissing style, when compared to the coaching style, was linked to more children’s negative emotions at the same time and was also connected to higher negative emotions and reduced emotion regulation over time. Except for these, dismissing style was also significantly associated with higher internalizing and externalizing problems at the same time.

### Definition of teachers’ meta-emotion philosophy

Alongside the well-established field of research focused on parental roles in children’s social–emotional competence, an increasing number of scholars have realized that teachers also provide essential socialization opportunities for children (Denham et al., 2012), develop emotional bonds with children in their classrooms (Ornaghi et al., 2019), spend considerable time with children, and perform numerous emotion-laden caregiving tasks (Ahn and Stifter, 2006). Various studies have indicated that teachers’ beliefs and attitudes can directly and indirectly impact children’s outcomes, such as their intelligence and academic abilities, self-perceptions, social reputation, and emotional knowledge (Coplan et al., 2015). Most research primarily concentrated on teachers’ beliefs and attitudes regarding children’s educational development, neglecting their beliefs and attitudes concerning their own and their students’ emotions, such as teacher’s meta-emotion philosophy (Ciucci et al., 2015; Hyson and Lee, 1996).

Furthermore, while there is a growing consensus that teachers, akin to parents, play a vital role in the socialization of children’s social–emotional competence (Morris et al., 2013), the distinctions between teachers and parents are also worthy of attention. For example, teachers always face a group setting and typically send socialization messages to children (Denham and Bassett, 2019). Consequently, contextual differences between teachers and parents might point to potentially different or unique contributions of teachers’ meta-emotion philosophy - the higher adult/child ratio in the classroom, for example, may dictate teachers a wider variety of meta-emotion approaches (Denham and Bassett, 2019). Teachers might adopt a coaching style as the dominant reaction to a child’s negative emotions, but then they have to turn to a dismissing style to effectively manage class group activities.

Ciucci et al. (2015) and Ciucci and Baroncelli (2024) first extended the construct of parental meta-emotion philosophy, respectively, to crèche educators and to teacher samples, drawing upon bodies of theory or research regarding parental meta-emotion philosophy (Eisenberg et al., 1998; Gottman et al., 1996), parenting self-efficacy (Bornstein, 2002), and teacher’s beliefs about emotion (Hyson and Lee, 1996). There were at least three core principles underlying teachers’ meta-emotion philosophy: (1) the practices they consider pertinent to their professional role; (2) the feeling of being able to implement these practices (i.e., self-efficacy); (3) their perception about the impact of their behaviors on children’s emotional development. In terms of children’s emotions, teachers’ meta-emotion philosophy is comprised of coaching and dismissing styles, self-efficacy as emotional socializer, and teachers’ perceived impact. Coaching and dismissing styles reflect teachers’ behavioral tendencies toward recognizing, accepting, and regulating children’s emotions. Teachers’ self-efficacy as emotional socializer refers to teachers’ perceived ability to carry out tasks associated with the role of emotional socializers. Teachers’ perceived impact refers to teachers’ evaluation of the effect of their behavior on the children’s emotional development. In terms of teachers’ own individual emotions, teachers’ meta-emotion philosophy comprises awareness, acceptance, and regulation of their own emotions. Emotion awareness is labeled by the ability to talk regarding and to differentiate emotions. Emotion acceptance refers to the meaning and value attributed to the emotions. Emotion regulation refers to the ability to control the strength and quality of emotions.

### Measures of teachers’ meta-emotion philosophy

According to the original definition of teachers’ meta-emotion philosophy above, Ciucci et al. (2015) developed the original Crèche Educator Emotional Style Questionnaire (CEESQ) to test the psychometric properties of a self-reported rating scale for early childhood teachers’ meta-emotion philosophy. It consisted of 36 items (i.e., 21 items rating teachers’ behaviors and beliefs about children’s emotions and 15 items rating teachers’ behaviors and beliefs about their own emotions). Participants were asked to rate each statement on a five-point from 1 (rarely or never) to 5 (very often). After exploratory and confirmatory factor analyses among 306 early childhood teachers, there remained 32 items and five dimensions: Coaching style (seven items, i.e., teachers’ awareness and acceptance of children’s emotions, and beliefs about the impact of early childhood teachers’ role in children’s emotional development), Dismissing style (five items, i.e., teachers’ lack of acceptance of children’s emotions), and teachers’ Self-efficacy as Emotional Socializers (six items, i.e., teachers’ beliefs about their abilities to deal with children’s emotions), Emotional Self-efficacy (10 items, i.e., teachers’ awareness and ability to deal with their own emotions), and Denial of Emotion (four items, i.e., teachers’ lack of acceptance of their own emotions). The former three were aimed at children’s emotions, and the latter two were aimed at teachers’ own emotions. Cronbach’s alpha for these dimensions ranged from 0.59 to 0.86. The CEESQ demonstrated equivalences in structure considering teachers’ parenting experiences, amount of teaching experiences, and academic education level.

Given that there is growing evidence that teachers’ emotional intelligence, beliefs, and personal values motivate and drive their professional self-efficacy and behaviors toward students in various school levels (Biesta et al., 2015; Jennings and Greenberg, 2009; Wu et al., 2019), Ciucci and Baroncelli (2024) thought all these constructs aforementioned were very similar to the meta-emotion philosophy and tried to apply the CEESQ among teachers from kindergarten to middle school. The main modification pertained to rewording “child” as “student” and “children” as “students.” Meanwhile, since many items had no participants’ responses in the extreme values of the 5-point Likert scale, the original scores were rescored on 3-point Likert scale. After confirmatory factor analyses among 815 female teachers from kindergarten to middle school, one item was removed from the final analyses due to low factorial loading, and the remaining 31 items supported the original five-factor structure of the CEESQ. The Cronbach’s alpha for these five dimensions above ranged from 0.62 to 0.90. Moreover, the CEESQ also demonstrated equivalences in structure considering school level, years of working experience, and academic education level.

### Applications of teachers’ meta-emotion philosophy

Since the establishment of the CEESQ, it has been extensively utilized, primarily encompassing the following four aspects. Firstly, some studies have investigated the influence of teachers’ personal characteristics on their meta-emotion philosophy. For example, Ciucci et al. (2015) found no main or interaction effects pertaining to teachers’ parenting experiences, years of working experience, and academic education level emerged in any of meta-emotion philosophy dimensions. Ornaghi et al. (2019) hypothesized the positive associations with teaching experience and meta-emotion philosophy. Unexpectedly, length of teaching experience was associated with low scores on the emotion-coaching scale, and higher scores in dismissive attitudes toward children’s emotional displays. All these provided additional supports that the CEESQ demonstrated equivalences in structure considering teachers’ personal characteristics, including parenting experiences, school level, years of working experience, and academic education level.

Secondly, based on a strict bond between how teachers recognize, accept, and regulate their own and their students’ emotions and the strong inter-relationships of the CEESQ dimensions, some studies tested whether self-efficacy as an emotional socializer could be a mediator between emotional self-efficacy and coaching styles (Ciucci and Baroncelli, 2024; Ciucci et al., 2015). In fact, both emotional self-efficacy and self-efficacy as emotional socializer were positively associated with a coaching style, with self-efficacy as emotional socializer playing a partial mediation role. This finding was consistent with the emotional connections between children’ and parents’ emotions (Gottman et al., 1996) and might provide new insights to explore the connections between teachers’ professional and individual emotional worlds.

Thirdly, several studies have investigated the associations between teachers’ emotional beliefs and meta-emotion philosophy. Ornaghi et al. (2019) found that early childhood educators’ “positive” beliefs about emotions were significantly correlated with a coaching emotion socialization style, while educators who thought that children should be protected from experiencing negative emotions obtained higher scores for the dismissing emotion socialization style. At the same time, there were significant relationships between teachers’ mind-mindedness and both “positive” beliefs about emotion and a coaching emotion socialization style. Ornaghi et al. (2021) found that educators’ coaching style was positively related to teaching about emotions, open expressiveness of one’s own emotional states, and beliefs regarding to the value of talking with children about emotions; Conversely, dismissing style was associated with beliefs concerning low merit of talking with children about emotional states and low teaching about emotion expression, as well as high protection of children from distressing emotions. All these findings provided additional supports that teachers’ emotional beliefs and personal values could motivate and drive their professional emotional characteristics.

Fourthly, certain research have focused more attention to the effects of meta-emotion philosophy on teachers’ emotion socialization practices. For instance, Ciucci et al. (2017) found early childhood teachers’ meta-emotion philosophy might enhance teacher-parent/colleague communication about children’s emotions. Ojala (2020) found senior high-school teachers’ views and beliefs about students’ emotions would influence the way how they perceive and deal with these emotional reactions during class teaching about climate change. By observing and coding early-childhood teachers emotion socialization practices in structured experimental situations at day care centre, Ornaghi et al. (2021) found that coaching reactions were the most frequently displayed by the teachers, followed by dismissing responses. Meanwhile, teachers’ coaching practices as observed in the experimental situations were positively associated with their self-perceived coaching style as assessed by the CEESQ. These results indicated that teachers’ social–emotional practices were frequently shaped by the underlying meta-emotion philosophy.

### Kindergarten teachers’ emotional socialization in China

Considering that early childhood is an essential stage for individual social–emotional competence development, promoting children’s social–emotional competence development has always been one of the crucial goals and main contents of early childhood education in China. For instance, Early Learning and Development Guidelines for Children Aged 3–6 years have listed “emotionally stable and Joyous,” “like to communicate with others,” and “enjoy and adapt to group life” as children’s learning and development goals (Ministry of Education, 2012). Nevertheless, some Chinese preschoolers are experiencing social–emotional difficulties, which hinder their future social and academic development (Huang and Siraj, 2023). Several results have indicated that the proportion of Chinese preschool children who have difficulties in their social–emotional competence was from 10% to 43.09% (Shen et al., 2021; Zhang et al., 2018). Among 2,363 children aged three to six from Shanghai, Li et al. (2023) found children’s social–emotional competence was generally positive, but there were significantly disproportionate across their gender, parental education level, etc. It is of practical significance to explore the factors that influence the development of children’s social–emotional competence.

Recently, domestic scholars have started to focus on the role of teachers in facilitating the social–emotional development of young children. For example, Zhang (2019) developed a foundational framework of social–emotional competence for kindergarten teachers, encompassing good teacher-student relationship, good relations of cooperation, self-development, and emotional management skills. Wu (2023) examined the effect of kindergarten teachers’ social–emotional competence on young children’s sociality. Shen and Wang (2022) explored how kindergarten teachers implemented social–emotional learning curriculum effectively. Ma et al. (2024) found that online mindfulness intervention course significantly improved the social–emotional competence of kindergarten teachers in the intervention group, while also dramatically reducing their job burnout levels. Recognizing the pivotal role of kindergarten teachers in facilitating children’s emotional development, Chang (2024) conducted a systematic review of pertinent international research and proposed that future studies should enhance investigations into internal mechanisms, standardize measurement, and promote practical applications. The research related to kindergarten teachers’ emotion socialization is becoming more and more abundant, however, the focus on Chinese kindergarten teachers’ underlying thoughts and beliefs, such as meta-emotion philosophy, that fuel their emotional behaviors and competence was revelatory. The main reason might be the absence of comparative measures. To fill this specific gap of the CEESQ not being validated among Chinese kindergarten teachers, the current study aimed to evaluate the psychometric properties of the CEESQ in the context of China.

### The current study

The main purpose of this study was to give additional support for previous findings by verifying the factor structure and convergent validity of CEESQ among Chinese kindergarten teachers. This was a multistage research consisting of two studies. According to suggestions from Ferrari et al. (2017), Study One involved two steps. First, exploratory factor analyses (EFAs) were conducted in Subgroup One to explain the largest amount of variance with each factor. Second, Cronbach’s alpha coefficients were provided for each factor, and Pearson correlation analyses were also conducted among these factors. According to suggestions from Zhu et al. (2024), Study Two involved three steps. First, confirmatory factor analyses (CFAs) were conducted to confirm the factor structure of CEEQS from Study One among a new independent sample (Subgroup Two). Second, measurement invariance (MI) was examined across teachers’ genders. Third, given the high interrelatedness across kindergarten teachers’ emotional intelligence and beliefs (Ciucci et al., 2017; Denham, 2023), preschool teachers’ emotional intelligence was evaluated to examine the convergent validity of their meta-emotion philosophy. Pearson correlation analyses were conducted among preschool teachers’ emotional intelligence and meta-emotion philosophy.

## Methods

### Participants

A convenient sampling method was applied in the current study. All data were collected through self-report questionnaires in *wjx.cn*, a reliable Chinese online platform. Informed consent was placed on the front page. Participants were free to decide whether to participate or not. We set the same IP, which could only be accessed once. Through this setting, we tried to ensure there is no any overlap in our sample. A total of 1,238 participants were recruited online. Excluding cases of invalid answers (e.g., continuous use of an option, too short or too long answering time, etc.), the number of retained participants was 1,114. Meanwhile, due to the low sample size, 41 male kindergarten teachers were also not included. Consequently, the final number of participants was 1,073. In order to conduct factor analysis, the current study randomly split the entire sample into two halves, with performing the EFA on Subgroup One and the CFA on Subgroup Two (Lorenzo-Seva, 2022). Table 1 presents pertinent demographic information for Subgroup One (*n* = 535) and Subgroup Two (*n* = 538).

**Table 1:** Demographic characteristics of preschool teachers.

Variables	Categories	Subgroup one	Subgroup two
Parenting experience	Having owned at least one child	322 (60.2%)	324 (60.2%)
	Having no child	213 (39.8%)	214 (39.8%)
Amount of teaching experiences	Below 10 years	352 (65.8%)	380 (70.6%)
	10 years or above	183 (34.2%)	158 (29.4%)
Academic education level	Below bachelor degree	150 (28%)	155 (28.8%)
	Bachelor degree or above	385 (72%)	383 (71.2%)

### Procedures

First, the CEESQ was translated and back-translated by two professional translators following the requirements provided by the International Test Commission (2005). Second, they compared their results to the original English version and pointed out any differences. Third, after making the necessary revisions, we invited ten postgraduate students with psychology and preschool education majors to rate the degree to which each scale item represented the objective or domain on a four-point scale. The Content Validity Index (CVI) test found that CVI for each item (I-CVI) ranged from 0.80–1.00 and CVI for the whole measure (S-CVI/Ave) was 0.97. According to the criteria (Lynn, 1986), our result indicated the Chinese version of CEESQ was with an excellent content validity. See specific items of the Chinese version of CEESQ in Table 2.

**Table 2:** Item factor loadings for the Chinese version of CEESQ.

Items	Factor one	Factor two	Factor three	Factor four	Factor five
When a student is angry, I help him/her to express what made him/her so angry 当一个学生生气时，我会帮助他/她表达是什么让他/她如此生气	0.85	0.09	0.06	−0.01	−0.11
When a student is feeling a negative emotion, it’s an opportunity to use my educational skills. 当一个学生感受到消极情绪时，这是我使用教育技能的机会	0.83	0.01	−0.05	−0.04	0.11
I try to change the negative mood of a student into a cheerful one. 我尝试把一个学生的消极的心情转变成欢乐的心情	0.77	0.01	0.05	0.03	0.18
When a student is happy, I take some time to share this feeling with him/her. 当一个学生高兴时，我会花一些时间同他/她分享这种感觉	0.75	0.03	0.03	−0.06	0.03
Students’ sadness is an emotion worth exploring. 学生的难过情绪是值得探究的	0.75	0.01	−0.11	0.06	0.01
When a student is afraid, I try to distract him/her. 当一个学生害怕时，我尝试分散他/她的注意力	0.72	−0.03	0.10	0.07	−0.04
The contribution of teachers to the emotional development of students is fundamental at school. 幼儿园教师对幼儿情绪发展发挥基础性作用	0.69	−0.01	0.11	0.03	0.01
When a student is angry, it’s an opportunity for getting close. 当一个学生生气时，这是接近他\她的好机会	0.65	−0.05	0.18	0.03	−0.03
I accept students’ fear even if it seems unmotivated. 我接受学生的畏惧，即使它似乎无缘无故	0.63	0.03	0.03	0.12	0.02
I can describe the strategies I use in order to cope with negative emotions. 我能描述出我用来应对消极情绪的策略	0.03	0.84	−0.01	0.07	0.07
When I am sad, I feel I can deal with the sadness I feel. 当我难过时，我觉得我能处理这种难过的情绪	−0.02	0.83	0.04	0.07	−0.08
I am able to prevent my fears taking over. 我能阻止畏惧蔓延开来	−0.09	0.82	0.01	0.04	0.02
I willingly accept the emotions I feel. 我愿意接受自己所感受到的情绪	0.00	0.82	−0.04	0.05	0.03
I feel in control of my emotions. 我觉得我可以控制自己的情绪	0.04	0.82	0.02	−0.10	0.11
I am able to manage emotions that are too intense. 我可以管理高强度的情绪	−0.03	0.75	0.17	−0.04	0.01
I am able to express what I feel. 我能表达自己所感受到的情绪	0.09	0.74	−0.03	−0.01	0.03
When I am angry, I have some control over my emotions. 当我生气时，我可以控制我的情绪	0.14	0.63	0.04	−0.18	−0.01
I can easily identify the reasons for my emotions. 我能很轻松地识别自己情绪的原因	0.07	0.63	(0.06)	0.07	−0.09
I feel able to help students cope with their fears and their anger. 我觉得自己可以帮助学生们处理他们的畏惧和生气	−0.03	0.07	0.79	−0.01	0.01
I can easily distinguish the different emotions a student is feeling. 我能很轻松地区别学生正在感受的不同情绪	0.01	0.08	0.76	−0.02	−0.04
I am able to stay close to an angry student. 我可以靠近一个生气的学生	0.07	−0.02	0.73	−0.10	0.02
I easily recognize the emotions that a student is experiencing. 我能很轻松地识别一个学生当下的情绪	−0.03	0.03	0.70	−0.02	0.17
I can get the students to express all of their emotions. 我可以让学生表达他们所有的情绪	0.01	0.11	0.66	0.01	0.02
When a student is angry, my goal is to make him/her stop. 当一个学生生气时，我的目标是制止他/她生气	0.01	0.01	−0.07	0.97	0.02
I help students get over sadness quickly so they can move on. 我帮助学生们尽快克服悲伤赶紧翻篇	0.01	0.03	−0.06	0.96	0.02
When I feel euphoric, I have the feeling of losing control. 当我感到欣喜时，我有失控的感觉	−0.13	−0.01	0.05	0.05	0.81
I feel uncomfortable when I do not have control over my emotions. 当我不能控制自己的情绪时，我觉得不舒服	0.10	0.07	−0.09	−0.09	0.76
I do not like the emotions I experience. 我不喜欢我感受到的情绪	0.09	−0.08	0.23	0.23	0.58
I perceive my negative emotions as something to defend myself against. 我把自己的消极情绪视为要抵御的东西	0.02	0.18	−0.11	−0.10	0.51

All these item factor loadings were from the final parallel EFA analysis.

### Measures

#### Demographic information

According to suggestions from Ciucci and Baroncelli (2024), kindergarten teachers’ individual parenting experience, amount of teaching experience, and academic education level were included. Kindergarten teachers’ parenting experience was measured by whether there was at least one child of their own. The amount of teaching experience was measured with “Below 10 years” or “10 years or above.” The reason for choosing 10 years of teaching instead of 12 years as the cut-off point was that many theories about teachers’ development cycles believed that 10 years of teaching was the dividing point between experienced and non-experienced teachers (Hargreaves, 2005). Academic education level was measured with “Below bachelor degree” or “Bachelor degree or above.” The reason for choosing a bachelor degree instead of high school as the cut-off point was that the latest data showed that the proportion of kindergarten teachers with college degrees or above was 87.8% (Ministry of Education, 2022).

#### Crèche educator emotional style questionnaire (CEESQ)

The teachers’ version of CEESQ consisted of 31 items and five expected factors: Coaching style (seven items, e.g., “Students’ sadness is an emotion worth exploring.”), Dismissing style (four items, e.g., “When a student is angry, my goal is to make him/her stop.”), Self-efficacy as emotional socializer (six items, e.g., “I can easily distinguish the different emotions a student is feeling”), Emotional self-efficacy (10 items, e.g., “I am able to prevent my fears taking over.”), and Denial of emotions (four items, e.g., “I perceive my negative emotions as something to defend myself against.”). Coaching style, dismissing style, and self-efficacy as emotional socializer involved teachers’ beliefs and behaviors concerning students’ emotions and teachers’ approaches toward students’ emotions. These factors were embraced in the CEESQ-Students’ Emotion section. Emotional self-efficacy and denial of emotions were teachers’ self-evaluations about their emotional competence. These factors were embraced in the CEESQ-Individual Emotion section. The response format was a three-point Likert scale (1 = “not true,” 2 = “sometimes true,” and 3 = “true”). Cronbach’s alpha for these five dimensions among Subgroup One and Subgroup Two will be reported in our results.

#### Emotional intelligence scale (EIS)

The EIS consisted of 16 items (Wong and Law, 2002). It embraced four expected factors: Self-emotion appraisal (four items, “I really understand what I feel”), Others’ emotion appraisal (four items, “I am a good observer of others’ emotions”), Uses of emotion (four items, “I always tell myself I am a competent person”), and Regulation of emotion (four items, “I have good control of my own emotions”). The response format was a seven-point Likert-type scale (from “1 = completely inconsistent” to “7 = completely consistent”). Cronbach’s alpha for these four dimensions ranged from 0.90 to 0.95 in Subgroup One and ranged from 0.92 to 0.95 in Subgroup Two, respectively.

### Data analyses

SPSS 22.0 was used to analyze the descriptive statistics, internal consistency, correlation analyses, convergent validity, and EFAs. Mplus 7.40 was used to conduct CFAs.

A principal factor analysis (PFA), with performing a Promax Rotation to allow for correlations between latent factors, was applied for EFAs. The final number of factor loading was based on eigenvalues (greater than one). The item selection was based on these criteria: (a) factor loading larger than 0.40 and (b) cross-factor loading lower than 0.30.

According to Cohen (1992), except for coaching style, other dimensions of the Chinese version of CEESQ and emotional intelligence did not violate the normal distribution (see Table 3). Thus, the maximum likelihood method (MLM) was applied for CFAs to examine the internal structure of the Chinese version of CEESQ. Further, measurement invariances of the Chinese version of CEESQ between various groups were empirically evaluated through a multigroup CFA to examine configural invariance (i.e., M1: identical structure across groups), metric invariance (i.e., M2: equivalent factor loadings across groups), scalar invariance (i.e., M3: identical intercepts, error variances, and covariances across groups), and residual invariance (i.e., M4: the sum of specific variance and error variance across groups) (Zhu et al., 2024). Muti-criteria, such as *χ^2^,* the comparative fit index (CFI), the Tucker-Lewis index (TLI), and the standardized root mean square residual (RMSEA), were included. To compare different models, we followed the suggestion from Cheung and Rensvold (2002) that changes in CFI (△CFI) and TLI (△TLI) were less than 0.01, indicating invariance between the different models.

**Table 3:** Descriptions, correlations, and Cronbach’s alpha coefficients.

Variables	Skewness	Kurtosis	M ± SD	Cronbach’s *α*	1	2	3	4	5
1 Coaching style	(−1.62) –1.88	(2.83) 3.10	(2.82 ± 0.31) 2.79 ± 0.33	(0.88) 0.89	1	–	–	–	–
2 Dismissing style	(−0.15) –0.11	(−048) -0.45	(2.08 ± 0.81) 2.06 ± 0.80	(0.93) 0.95	(0.20^**^) 0.19^**^	1	–	–	–
3 Self-efficacy as Emotional Socializer	(−0.57) –0.97	(0.52) 0.69	(2.83 ± 0.33) 2.79 ± 0.37	(0.86) 0.89	(0.86^**^) 0.84^**^	(0.17^**^) 0.15^**^	1	–	–
4 Emotional Self-efficacy	(−1.18) –1.08	(0.77) 0.29	(2.64 ± 0.44) 2.63 ± 0.45	(0.92) 0.92	(0.57^**^) 0.54^**^	(0.22^**^) 0.15^**^	(0.60^**^) 0.59^**^	1	–
5 Denial of Emotions	(−0.09) 0.01	(−0.62) –0.53	(1.91 ± 0.53) 1.88 ± 0.51	(0.65) 0.64	(−0.29^**^) –0.20^**^	(−0.30^**^) –0.21^**^	(−0.24^**^) –018^**^	(−0.27^**^) –0.24^**^	1
6 Self-emotion appraisal	(−1.02) –0.93	(0.75) 0.42	(6.09 ± 1.02) 6.06 ± 1.02	(0.95) 0.95	(0.47^**^) 0.49^**^	(0.18^**^) 0.14^**^	(0.48^**^) 0.50^**^	(0.61^**^) 0.57^**^	(−0.10^*^) –0.06^*^
7 Others’ emotion appraisal	(−0.63) –0.60	(−0.04) –0.21	(5.82 ± 1.05) 5.85 ± 1.09	(0.90) 0.93	(0.46^**^) 0.46^**^	(0.23^**^) 0.18^**^	(0.49^**^) 0.45^**^	(0.53^**^) 0.50^**^	(−0.20^**^) –0.13^**^
8 Uses of emotion	(−0.80) –0.78	(0.22) 0.20	(5.96 ± 1.04) 5.87 ± 1.09	(0.91) 0.92	(0.47^**^) 0.47^**^	(0.23^**^) 0.15^**^	(0.47^**^) 0.48^**^	(0.63^**^) 0.64^**^	(−0.12^**^) –0.09^*^
9 Regulation of emotion	(−0.74) –0.82	(−0.02) 0.55	(5.82 ± 1.14) 5.83 ± 1.12	(0.94) 0.95	(0.45^**^) 0.45^**^	(0.23^**^) 0.14^**^	(0.47^**^) 0.46^**^	(0.69^**^) 0.68^**^	(−0.09^*^) –0.08^*^

The numbers in parentheses were from Subgroup One. ^*^ *p* < 0.05. ^* *^p < 0.01.

## Results

### Step 1: exploratory factor analysis for the Chinese version of CEESQ

The Kaiser–Meyer–Olkin value of 0.93, which was above 0.60, and Bartlett’s test of sphericity approximate, *χ^2^*(406) = 10215.53; *p* < 0.001, indicating that the data was factorable for the factor analysis. The result revealed that five factors were extracted based on eigenvalues (greater than 1). The item factor loadings are detailed in Table 2. Importantly, the item-“When my mood changes I easily recognize my emotion” and the item-“I feel I am very good at making the children reflect on what made them angry, frightened or sad” did not load strongly onto any of the five factors (item factor loading <0.30). The principal component analyses and parallel analyses were repeated twice. After excluding these two items one by one, the final parallel analysis also suggested that five factors should be extracted from the Chinese version of CEESQ. All these five factors could be accounted for 62.65% of the total variance. Factor One included nine items and was labeled “Coaching style.” It accounted for 37.55% of the variance, and its factor loadings ranged from 0.63 to 0.85. Factor Two included nine items and was labeled “Emotional self-efficacy.” It accounted for 8.95% of the variance, and its factor loadings ranged from 0.63 to 0.84. Factor Three included five items and was labeled “Self-efficacy as emotional socializer.” It accounted for 7.84% of the variance, and its factor loadings ranged from 0.66 to 0.79. Factor Four included two items and was labeled “Dismissing style.” It accounted for 4.99% of the variance, and its factor loadings ranged from 0.96 to 0.97. Factor Five included four items and was labeled “Denial of emotions.” It accounted for 3.32% of the variance, and its factor loadings ranged from 0.51 to 0.81. Moreover, Pearson correlation analyses indicated that all these variables were correlated with each other (*ps* < 0.01, see Table 3).

### Step 2A: confirmatory factor analyses for the Chinese version of CEESQ

The CFA with 29 items revealed that the five-factor model (Model 1) fitted well, with *χ^2^/df* = 3.70, CFI = 0.93, TLI = 0.92, RMSEA = 0.061 (90% CI = [0.057, 0.065]), RMSEA = 0.05. Moreover, we also constructed other three increasingly complex models: a five-factor model consistent with the original questionnaire (Model 2, *χ^2^/df* = 5.44, CFI = 0.85, TLI = 0.84, RMSEA = 0.091 90% CI = [0.087, 0.095], RMSEA = 0.15), a single-factor model (Model 3, *χ^2^/df* = 11.45, CFI = 0.65, TLI = 0.62, RMSEA = 0.139 90% CI = [0.136, 0.143], RMSEA = 0.07), and a five-factor model reflecting the two different CEESQ sections (Model 4, *χ^2^/df* = 15.38, CFI = 0.52, TLI = 0.48, RMSEA = 0.163 90% CI = [0.160, 0.167], RMSEA = 0.10). Comparisons of these models with the five-factor model did not fit the data better.

### Step 2B: measurement invariance of the Chinese version of CEESQ

All configural invariance models showed an acceptable fitness to the data, indicating that kindergarten teachers with/without children, with/without more than 10 years of teaching experience, and with/without a bachelor degree conceptualized the construct similarly.

After configural invariance models were confirmed, the metric, scalar, and residual models were also found to be equivalent across kindergarten teachers with/without children, with/without more than 10 years of teaching experience, and with/without a bachelor degree, according to the following fit indices: △CFI ≤ 0.01 and △TLI ≤ 0.01 (see Table 4). These results suggested the measurement invariance of the Chinese version of CEESQ across various Chinese kindergarten teachers.

**Table 4:** Results of MI across multiple groups among Subgroup Two.

Model	χ^2^	df	χ^2^/df	CFI	TLI	RMSEA [90% CI]	SRMR	△CFI	△TLI
MI across “Parenting experience”									
Configural	1845.94	628	2.94	0.920	0.912	0.065 [0.060 0.069]	0.056	–	–
Metric	1880.54	650	2.89	0.919	0.912	0.064 [0.059, 0.068]	0.057	−0.001	0.000
Scalar	1937.60	672	2.88	0.917	0.913	0.068 [0.065, 0.071]	0.061	−0.002	0.001
Residual	2146.94	683	3.14	0.913	0.910	0.069 [0.065, 0.074]	0.074	−0.004	−0.003
MI across “Amount of teaching experience”									
Configural	1884.53	628	3.00	0.918	0.911	0.069 [0.066, 0.072]	0.047	–	–
Metric	1931.71	650	2.97	0.920	0.913	0.069 [0.065, 0.074]	0.058	−0.001	0.002
Scalar	1982.48	672	2.95	0.915	0.914	0.068 [0.065, 0.071]	0.066	−0.005	0.001
Residual	2024.77	683	2.96	0.908	0.905	0.071 [0.069, 0.074]	0.077	−0.007	−0.009
MI across “Academic education level”									
Configural	1820.67	628	2.90	0.922	0.919	0.064 [0.060, 0.069]	0.053	–	–
Metric	1844.73	650	2.84	0.920	0.918	0.063 [0.058, 0.067]	0.064	−0.002	−0.001
Scalar	1864.42	672	2.77	0.912	0.917	0.065 [0.062, 0.068]	0.066	−0.008	−0.001
Residual	1887.48	683	2.76	0.908	0.918	0.071 [0.067, 0.075]	0.070	−0.004	0.001

MI, measurement invariance.

### Step 2C: convergent validity of the Chinese version of CEESQ

First, Cronbach’s alpha coefficient for kindergarten teachers’ meta-emotion philosophy dimensions ranged from 0.65 to 0.93 in Subgroup One and ranged from 0.64 to 0.95 in Subgroup Two, demonstrating acceptable internal consistency reliability. Second, all these dimensions were significantly correlated with emotional intelligence (*ps* < 0.05).

## Discussion

Following our multi-staged tests, this study provided additional support for the five-factor structure of CEESQ in our two Chinese kindergarten teacher samples. Despite consistency in the number of factors between the current sample and Italian samples (Ciucci and Baroncelli, 2024; Ciucci et al., 2015), the specific items of factors across these samples were slightly various in the following two aspects.

First, two items (i.e., “I try to change the negative mood of a student into a cheerful one” and “When a student is afraid, I try to distract him/her”) failed to load on the original Dismissing style but they loaded on Coaching style. These two items in the original version refer to the belief that children’s negative emotions are not worth exploring and teachers do not need to pay attention to them actively. Interestingly, Chinese kindergarten teachers perceived these two items as the Coaching style, which referred to teachers’ recognition, acceptance, and regulation of children’s emotional expression. One possible explanation for this result might be the differences between collectivist and individualistic cultures (Fiorilli et al., 2015; van Hemert et al., 2007). In collectivist cultures, typical of Eastern societies, the mainstream values emphasize interdependence among individuals and group identity. To maintain social harmony and show respect for other group members’ emotions, caregivers in collectivist cultures always tend to socialize their children’s emotions by promoting children’s emotional self-regulation, limiting children’s expression of negative emotions, and developing children’s high levels of self-control (Mesquita and Frijda, 1992). Conversely, in individualist cultures, typical of Western societies, the leading values prioritize nurturing individual independence, personal assertion, and the accomplishment of personal goals (Hofstede, 2001). Consequently, caregivers in individualist cultures always encourage children’s emotional expressiveness and hold the belief that intra-personal and subjective experiences significantly contribute to defining individual feelings. In line with this distinction, when faced with a child’s negative emotions, while an Italian kindergarten teacher would encourage children to express and explore their emotions, a Chinese kindergarten teacher would be prone to change them into cheerful ones or distract children immediately to maintain positive and happiness in their activities. Through a class observation, Liang and Wang (2015) found that the main ways used by Chinese kindergarten teachers to regulate preschoolers’ negative emotions were giving commands to suppress their emotions, distracting their attention, and helping children vent their emotions. The rates of their occurrence were 51%, 30.4%, and 18.6%, respectively. Conversely, Italian teachers showed more supportive reactions to children’s emotions. With observations, Ornaghi et al. (2021) found that Italian teachers were usually supportive toward children’s emotions, and welcome the expression of both positive and negative emotions.

Second, due to low factorial loading, two items were removed from the final analyses. For the deletion of the item-“I feel I am very good at making the children reflect on what made them angry, frightened or sad,” the main reason was also related to the aims of Chinese early childhood education. With a textual analysis of Chinese early childhood policies from 1978 to 2019, Huang (2022) found that although the Chinese government has attached great importance to the cultivation of preschoolers’ social–emotional competence, most of these policies overly emphasize preschoolers’ emotional regulation and control and the binding force of “heteronomy” and “rules.” Consequently, teachers tend to focus on whether children can regulate their emotions quickly and rarely pay attention to the causes of children’s emotions. For the deletion of the item-“When my mood changes I easily recognize my emotion,” the main reason might be related to kindergarten teachers’ high level of job burnout. Confronted with heavy workloads, low social acceptance, and low personal psychological literacy, kindergarten teachers often face high levels of job burnout, which is typically characterized by emotional exhaustion. Among 1795 kindergarten teachers in Tianjin, China, Li et al. (2020) found the prevalence of burnout in Chinese kindergarten teachers was 53.2%, which was higher than the prevalence of burnout in other countries. With a meta-analysis, Huang et al. (2024) found the mental health problems of kindergarten teachers in China showed an upward trend from 1998 to 2020. In this regard, with a high level of job burnout, kindergarten teachers are prone to neglect their own emotions and fail to recognize their emotional changes.

Apart from those above, this study also provided valuable pieces of evidence demonstrating that the Chinese version of CEESQ had measurement invariance across various groups and adequate criterion validity among kindergarten teachers in China. In terms of the measurement invariance of the Chinese version of CEESQ, our results were consistent with the previous studies (Ciucci and Baroncelli, 2024; Ciucci et al., 2015). These indicated that teachers’ parenting experiences, amount of teaching experience, and academic education level did not influence the factor structure of kindergarten teachers’ meta-emotion philosophy. In terms of adequate criterion validity, our results also found that teachers’ meta-emotion philosophy was significantly correlated to their emotional intelligence. While limited has directly discussed the relationship between teachers’ meta-emotion philosophy and emotional intelligence, studies on the association teachers’ meta-emotional philosophy and their emotional socialization could provide a basis for this. For example, Ciucci et al. (2017) found that teachers with positive meta-emotional philosophy, such as emotional self-efficacy, self-efficacy as emotional socializer, and coaching style tend to be more willing to communicate children’s emotions with their parents and colleagues. The reason may be that when a teacher possesses positive meta-emotional philosophy, he/she is more likely to perceive and understand others’ feeling regarding the current issue during the dialogue. Accordingly, this teacher could expand her understanding of this context to use her emotional expressiveness to put her colleague or student at ease, through smiling and offering comfort. From this viewpoint, positive meta-emotion philosophy could help individuals in appraising, using, and regulating emotions. All of these are core of individuals’ emotional intelligence (Wong and Law, 2002). Thus, fostering teachers’ adaptive meta-emotion philosophy might promote their emotional competence, consequently yielding beneficial emotional socialization enacted in the classroom. Future research needs to explore their relationships comprehensively.

## Limitations and future directions

Our findings should be interpreted in light of the present study’s limitations. First, our main research design was cross-sectional, which could not provide more information about the longitudinal invariance of the Chinese version of CEESQ. Future research should explore the developmental characteristics of kindergarten teachers’ meta-philosophy through a longitudinal approach. Additionally, aside from coaching and dismissing styles, there may exist alternative emotional socialization styles among teachers. Ornaghi et al. (2021) discovered a previously unidentified emotional socialization style among teachers through a systematic observation. This style, known as amplification, primarily emphasized the inequality between the children and exacerbated their negative emotions. Cultural factors might influence caregivers’ emotional socialization. Individuals from collectivist cultures may be more inclined to express and manage their emotions through spiritual activities, allowing them to process challenging feelings and seek solace without distressing their loved ones (Daga et al., 2015; Merchant, 2024). Therefore, future research needs to broaden the framework of teachers’ meta-emotion philosophy, especially for the dismissing style, by applying mixed approaches, such as a qualitative interview or class observation. Third, consistent with the previous studies (Ciucci and Baroncelli, 2024; Ciucci et al., 2015), dismissing style was positively associated with coaching style, indicating that teachers might prefer one style instead of the other based on various situations. Kindergarten teachers might adopt a coaching style as the dominant reaction to children’s negative emotions, but then they have to turn to a dismissing style to effectively manage class activities. Future studies must further distinguish between teachers’ meta-emotion philosophy in response to a single child’s emotions and the whole class group’s emotional activation during daily interaction. Finally, the inadequate number of male kindergarten teachers in our entire sample precluded their inclusion in later analyses. However, it is essential to acknowledge the disparities in emotional socialization between male and female educators (Demetriou et al., 2009). Therefore, it may be advantageous to incorporate male educators in future research.

## Conclusion

In conclusion, the Chinese version of CEESQ is a promising instrument with sound reliability and validity for measuring meta-emotion philosophy among Chinese kindergarten teachers. First, the Chinese version of CEESQ can help kindergarten teachers understand how their emotional socialization influences children’s emotional development. Secondly, it adds to the existing research literature supporting the applicability of CEESQ with diverse cultural groups. However, further research is required to examine the replicability and psychometric properties of the Chinese version of CEESQ with longitudinal and mixed approaches and among various situations, such as a single student’s emotions and the whole class group’s emotional activities.

## Data Availability

The raw data supporting the conclusions of this article will be made available by the authors, without undue reservation.
